# Geometric Model and Numerical Study of Aqueous Humor Hydrodynamics in the Human Eye

**DOI:** 10.1155/2022/4756728

**Published:** 2022-04-11

**Authors:** Hao Tang, Zhangrong Qin, Binghai Wen

**Affiliations:** ^1^Guangxi Key Lab of Multi-Source Information Mining and Security, Guangxi Normal University, Guilin 541004, China; ^2^School of Computer Science, Guangdong University of Science and Technology, Dongguan 523083, China

## Abstract

The flow of aqueous humor (AH) in the human eye plays a key role in the process of transporting nutrients to the intraocular tissues and maintaining normal intraocular pressure. The pathogenesis of many ophthalmic diseases is also closely related to the flow of AH. Therefore, it is of great significance to study the mechanism of AH dynamics in the human eye. In this paper, we used image processing technology to denoise and segment the anterior segment optical coherence tomography (AS-OCT) images and established a geometric model based on the human eye. At the same time, a model of AH dynamics in the human eye based on the lattice Boltzmann (LB) method was proposed. Then, we simulated the AH flow in the human eye with different morphological structures and different physical properties and analyzed the factors that affect the AH flow, including the shape of anterior chamber (AC), the crypts of iris, the indentation of cornea, the permeability of trabecular meshwork (TM), the secretion rate of AH, and the viscosity of AH. The results showed that the changes in eye tissue morphological structures and physical properties would affect the flow of AH. For example, the maximum velocity of AH flow decreases with the increases in cornea deformation. When the distance of cornea indentation changes from 0.3 mm to 0.5 mm, the maximum velocity of AH reduces by 17%. In the asymmetrical AC, the AH will form two different vortices. In the crypts of the iris, we found that the AH flow forms small vortices, a phenomenon that has not been reported in other papers. In addition, we found that the intraocular pressure (IOP) decreases with the increase of the TM permeability and increases with the increase of the AH secretion rate, and it is not sensitive to changes in the viscosity of AH.

## 1. Introduction

The human eye is one of the most important sensory organs of the human body, and its structure is very complex. AH in the human eye plays a critical role in physiological processes such as delivering nutrients to the eye tissues and maintaining normal IOP [1]. In addition, AH is also closely related to the pathogenesis of many eye diseases, such as glaucoma and cataracts, which are two common blinding eye diseases [2–4]. As early as the 1850s, researchers used fluorescence technology to calculate the production rate of AH by injecting fluorescein from a vein and then measuring the rate at which fluorescein disappears in the AC [5]. Due to the complexity of the eye structure, some data cannot be directly measured through experiments [6]. Faced with these problems, scholars have carried out extensive research and achieved some important results.

To study the dynamics of AH in the human eye, the first step is to understand the factors that drive the flow of AH. Canning et al. [7] used a standard fluid dynamics model to study the flow of AH in the AC, and their results showed that the AH flow is mainly affected by the temperature difference of AC. Heys and Barocas [8] regarded AH as Boussinesq fluid, established a Boussinesq model of natural convection of AH in the human eye. Ooi and Ng [9] established a two-dimensional human eye model to study the temperature distribution of the AH flow. The results show that the flow of AH increases the heat transfer rate in the human eye and increases the temperature in the anterior segment (AS) but has no obvious effect on the posterior segment. Karampatzakis and Samaras [10] constructed a three-dimensional human eye heat transfer numerical model and studied the distribution of temperature under the flow of AH and found that the temperature distribution of the AC is very closely related to the flow of AH.

Another research focus of AH dynamics is the outflow process of AH from TM and Schlemm's canal (SC). TM and SC are the main outflow channels of AH. Once TM and SC become diseased, the outflow of AH will be blocked, resulting in increased IOP. High IOP is one of the main causes of glaucoma [5]. In open-angle glaucoma, the anterior chamber angle is always open, and the outflow of AH is obstructed by TM and SC, as shown in reference [11]. Merchant and Heys [12] studied the influence of different permeability of TM on the AH outflow and analyzed the effect of the TM structure on the obstruction of AH outflow and the increase of IOP in open-angle glaucoma. Guo et al. [13] established a model composed of TM and SC, studied the characteristics of AH flow velocity, streamline, and wall shear stress under different SC morphological parameters, and revealed the relationship between SC morphological parameters and AH dynamics. Nowadays, the research on TM and SC mostly focuses on the influence of their physical structure on the outflow resistance of AH. Therefore, the mechanical properties of TM and SC, such as elastic modulus, are the focus of many simulation studies.

Some scholars have studied the influence of ocular morphological structures. Zuhaila et al. [14] believed that the cornea indentation changes the structure of AC and makes the AH flow slow. Wang et al. [15] established a model based on the physiological mechanism of AH and the principle of fluid dynamics and analyzed the relationship between iris deformation and IOP using a fluid structure coupling method. Kumar et al. [16] believed that pupil size has little effect on the AH flow in the rabbit eye. Wang et al. [17] used particle image velocimetry to visualize the flow of AH and compared the difference between the flow of AH under normal conditions and pupil block conditions.

The above studies have greatly helped us understand the AH dynamics. However, many of the models used in studies are theoretical models that differ greatly from actual human eyes. There are differences in different eyes, such as cornea indentation affected by external force [14], iris bombe caused by cancer [18], and narrow AC angle in angle-closure glaucoma [11], which will have an important impact on the AH dynamics. Therefore, in the simulation, we must consider the influence of these factors on the numerical simulation results. In the study of Xu et al. [19], the shallow AC and the thick cornea are related to each other, and the thickness of cornea will take away some space from AC. Therefore, when performing related numerical simulations, simply increasing the thickness of cornea, ignoring its relationship with the depth of AC, will inevitably deviate from the real results. In the case of angle-closure glaucoma, the morphological changes caused by iris bombe are more pronounced and greatly different from the structure of normal human eye, as shown in reference [11]. In this case, the theoretical model based on anatomical structure data is no longer applicable. In addition, the fluid mechanic's methods used in most of these studies are based on the continuous medium hypothesis, like the finite element method, and commercial fluid simulation software is used for analysis. These methods have some shortcomings in dealing with the problems such as AH flow with complex structure and multiple physical processes.

The main research content of this paper is to use the AS-OCT images and image processing technology, combined with the structure data of human eye, to establish a more realistic AS geometric model; at the same time, use the LB method to establish a model of the AH dynamics. On this basis, the model is used to simulate and analyze the AH flow in the human eye with different morphological structures and different physical characteristics. We first conducted the numerical simulations of AH flow in the normal human eyes and studied the velocity and temperature distribution of AH in standing and lying positions. In terms of morphological structure, we selected factors including AC shape, cornea indentation, and iris crypts to analyze the influence of AS structure on AH flow. At the same time, we also studied the effect of TM permeability, AH secretion rate, and AH viscosity coefficient on IOP. In the study, we compared the theoretical geometric model and the geometric model based on AS-OCT images and also compared the conditions of normal eyes and pathological eyes.

The model of AH dynamics based on AS-OCT images and the LB method can more accurately reflect the flow of AH in the real human eye and obtain some physiological and pathological data that are difficult to directly measure through experiments. It can provide some numerical theoretical reference for further understanding of the physiological structure of the human eye and the pathogenesis of ophthalmic diseases.

## 2. Geometric Model

In the study of AH dynamics, the first task is to establish a geometric model that can accurately depict the shape and position of each intraocular tissue in the AS. In this section, we will extract the structural information of AS from the AS-OCT image to establish the geometric model of AS with individual characteristics and then apply it to the subsequent numerical simulation.

### 2.1. Image Denoising

The AS-OCT image is one of the applications of OCT in medical imaging. OCT is a technology that uses the weak coherence characteristics of light to perform high-resolution imaging of the internal tissue structure of organisms. [20] Due to its imaging mechanism, it will inevitably be polluted by noise in the process of acquiring images, which will affect the subsequent image processing. [21] Therefore, it is necessary to denoise the AS-OCT image.

In the AS-OCT image, the main type of noise is speckle noise; so, we select an anisotropic filter that has good denoising effect on speckle noise to complete the denoising of the image. The denoising model [22] is as follows:

(1) $$\begin{array}{c} \frac{\partial \rho \left(x,t\right)}{\partial t}=\frac{M\Delta h^{2}}{18\Delta t}\underset{i}{\sum }g_{i}\left(x\right){\left(e_{i}\cdot \nabla \right)}^{2}\rho \left(x,0\right)-\frac{M\Delta h}{9\Delta t}\underset{i}{\sum }g_{i}\left(x\right)\left(e_{i}\cdot \nabla \right)\rho \left(x,0\right), \end{array}$$

where *ρ*(*x*, 0) represents the original image, and *g*_*i*_(*x*) is the particle passing rate. The larger the image gradient is, the smaller the particle pass rate is and the slower the particle diffusion speed is, while the smaller the image gradient is, the larger the particle pass rate is and the faster the particle diffusion speed is. In this way, the edge information can be retained while denoising.

**Figure 1:**
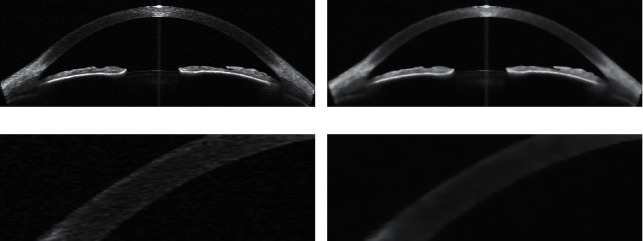
(a) An original AS-OCT image. (b) AS-OCT image after denoising. (c) An enlarged area of the original image. (d) An enlarged area of the denoised image.

Figure 1(a) shows an original AS-OCT image, as can be seen from the enlarged part of the image, Figure 1(c), and there is obvious noise in the background of the image, which will certainly interfere with subsequent image processing steps. Figures 1(b) and 1(d) show the results of denoising the AS-OCT image using the denoising algorithm. By comparing Figures 1(c) and 1(d), it can be found that most of the noise in the background of the original image is removed, but the key structural information in the image is still retained.

### 2.2. Image Segmentation

Because only the edge information of the AS-OCT image is used in geometric modeling, no other additional data is needed; so, this paper intends to adopt an image segmentation method based on edge detection in image processing. The edge detection algorithm based on the anisotropic filter is used here. Compared with other edge detection algorithms, this algorithm has the characteristics of accurate edge detection and good antinoise performance. The algorithm model [23] is as follows:

(2) $$\begin{array}{c} \frac{\partial \rho \left(x,t\right)}{\partial t}=\frac{M\Delta h^{2}}{3\Delta t}\left(\tau -\frac{1}{2}\right)\nabla^{2}\left[\rho \left(x,0\right)*G\left(x\right)\right], \end{array}$$

where *G*(**x**) is a Gaussian filter.

**Figure 2:**
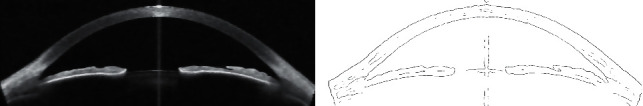
(a) The denoised AS-OCT image. (b) The result of edge detection.

Figure 2 shows the result of edge detection on the denoised AS-OCT image.

**Figure 3:**
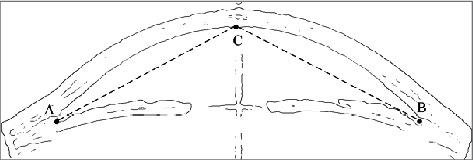
Extracting the structure information of intraocular tissues.

After obtaining the edge information of the image, we can extract the structural information of eye tissue from the edge image according to the needs of geometric modeling. For example, the contour of the cornea is extracted by specifying feature points in the image, as shown in Figure 3. According to the characteristics of the AS-OCT image, manually select the three points *A*, *B*, and *C*, traverse the edge points in the area above the line segment AC and line segment BC from bottom to top, and record the first edge point found in each column, which is the cornea edge point.

### 2.3. Building the Flow Field

Further processing is needed after obtaining the feature information of the AS-OCT image. Due to the limitation of the imageable area of the AS-OCT image, the image cannot display the structure of the posterior chamber (PC) and other intraocular tissues. Therefore, we need to reestablish the structure of PC and other intraocular tissues based on the ocular tissue data [24–28].

We first extend downward from the intersection of the cornea and the iris, draw the PC with a height of 0.5 mm as the entrance to the AH, then draw the AS length of 12.8 mm, and then draw the lens with a curvature radius of 10 mm based on the pupil axis, so as to complete the construction of the PC and other tissues.

**Figure 4:**
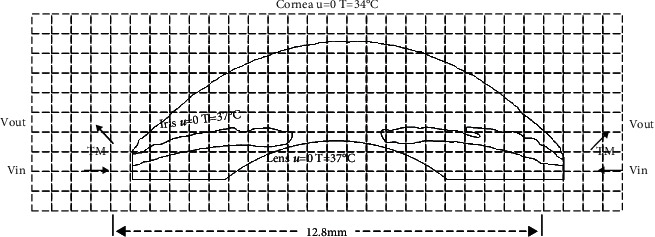
The geometric model based on the AS-OCT image.

In order to enable the geometric model to be used in subsequent numerical simulations and generate the flow field suitable for the LB method, type annotation and meshing are also required. According to the dimensional transformation, there are about 500,000 grid points in total.  Figure 4 shows the final geometric model result. In the model, the surface of cornea, iris, and lens are set with rigid no-slip boundary with the velocity of 0, and the velocity boundary conditions are applied at TM and AH inlet. The temperature of cornea is set to 34°C, and the temperature of AH and other intraocular tissues is set to 37°C [28].

## 3. AH Dynamic Model

### 3.1. Macroscopic Equations

The main component of AH is water, which also contains a small amount of protein and other substances [29]. Its physical properties are very close to water [30], and we regard it as a Newtonian fluid; so, we use the incompressible Navier-Stokes (*N*-*S*) equation to describe the AH flow:

(3) $$\begin{array}{c} \nabla \cdot u=0, \end{array}$$

(4) $$\begin{array}{c} \frac{\partial u}{\partial t}+\nabla \cdot \left(uu\right)=-\nabla p+v\nabla^{2}u+F. \end{array}$$

Because of the temperature difference in the tissues of the eye, we must consider the heat transfer process. For the flow with temperature changes, if the viscous heat dissipation and the work done by pressure are neglected, the temperature can be regarded as a passive scalar that moves with the fluid and conforms to a convection diffusion equation:

(5) $$\begin{array}{c} \frac{\partial T}{\partial t}+\nabla \cdot \left(uT\right)=\theta \nabla^{2}T. \end{array}$$

In addition, we use Darcy's law to describe the process of AH flowing out of TM:

(6) $$\begin{array}{c} u=-\frac{K}{\mu }\nabla p. \end{array}$$

For the three coexisting physical processes of AH flow, heat transfer, and outflow of the TM, we can use Boussinesq approximation to complete the coupling between AH flow and heat transfer in AS. Under the condition of Boussinesq approximation, in addition to the density of the external force, the physical properties of the fluid, such as density, viscosity coefficient, and thermal diffusion coefficient, are all regarded as constants. At the same time, the density in the external force has the following form:

(7) $$\begin{array}{c} \rho =\rho_{0}\left[1-\beta \left(T-T_{0}\right)\right]. \end{array}$$

From this, we can calculate the external force:

(8) $$\begin{array}{c} F=-g\beta \left(T-T_{0}\right). \end{array}$$

Through Equations (3)-(8), the coupling between the AH flow and heat transfer in the AS can be completed, and the equations based on Boussinesq approximate coupling can be obtained:

(9) $$\begin{array}{c} \begin{array}{c} \nabla \cdot u=0,\\ \frac{\partial u}{\partial t}+\nabla \cdot \left(uu\right)=-\nabla p+v\nabla^{2}u-g\beta \left(T-T_{0}\right),\\ \frac{\partial T}{\partial t}+\nabla \cdot \left(uT\right)=D\nabla^{2}T. \end{array} \end{array}$$

For the coupling between the flow of AH in eye and the outflow of AH from TM, we can calculate the IOP from the incompressible N-S equation, then solve the pressure gradient at TM, and then calculate the seepage velocity from Darcy's law according to the pressure gradient. At last, we applied the calculated velocity to the incompressible N-S equation. Thus, the coupling between two processes is completed.

### 3.2. LB Model

The entire AS area can be regarded as a flow field. In this flow field, there is a velocity field formed by the flow of AH and a temperature field formed by the temperature difference between intraocular tissues. Therefore, it is necessary to use two independent LB equations to describe the field of velocity and temperature the separately and then use Boussinesq approximation to unify the two LB equations into a whole system, so as to complete the solution of the macro equation.

We use the D2G9 model [31] to simulate the velocity field, and its evolution equation is as follows:

(10) $$\begin{array}{c} f_{i}\left(x+e_{i}\Delta t,t+\Delta t\right)=f_{i}\left(x,t\right)-\frac{1}{\tau }\left[f_{i}\left(x,t\right)-f_{i}^{\text{eq}}\left(x,t\right)\right]+F_{i}, \end{array}$$

where *f*_*i*_ is the particle distribution function, *f*_*i*_^eq^ is the equilibrium distribution function, *e*_*i*_ is the discrete velocity, and *τ* is the dimensionless relaxation time. The equilibrium distribution function is set as follows:

(11) $$\begin{array}{c} f_{i}^{\text{eq}}\left(x,t\right)=\left\{\begin{array}{c} -4\sigma \frac{p}{c^{2}}+S_{0}\left(u\right),i=0,\\ \lambda \frac{p}{c^{2}}+S_{i}\left(u\right),i=1,2,3,4,\\ \gamma \frac{p}{c^{2}}+S_{i}\left(u\right),i=5,6,7,8, \end{array}\right. \end{array}$$

(12) $$\begin{array}{c} u=\overset{8}{\underset{i=1}{\sum }}{ce}_{i}f_{i}, \end{array}$$

(13) $$\begin{array}{c} p=\frac{c^{2}}{4\sigma }\left[\overset{8}{\underset{i=1}{\sum }}f_{i}+S_{0}\left(u\right)\right]. \end{array}$$

The LB equation for simulating the temperature field is as follows:

(14) $$\begin{array}{c} T_{i}\left(x+{\text{e}}_{i}\Delta t,t+\Delta t\right)=T_{i}\left(x,t\right)-\frac{1}{\tau_{T}}\left[T_{i}\left(x,t\right)-T_{i}^{\text{eq}}\left(x,t\right)\right], \end{array}$$

where *τ*_*T*_ is the relaxation time of the temperature field, and *T*_*i*_^eq^ is the equilibrium distribution function of the temperature field. *T*_*i*_^eq^ is set as follows:

(15) $$\begin{array}{c} T_{i}^{\text{eq}}\left(x,t\right)=\frac{T}{4}\left[1+2\frac{e_{i}\cdot u}{c}\right]. \end{array}$$

The macroscopic temperature *T* of AH can be calculated from the distribution function of temperature field:

(16) $$\begin{array}{c} T=\overset{4}{\underset{i=1}{\sum }}T_{i}\left(x,t\right). \end{array}$$

In order to complete the coupling of the velocity field and the temperature field, it is necessary to use Boussinesq approximation; that is, the external force in equation (10) is obtained by equation (8), and its discretization form is

(17) $$\begin{array}{c} F_{i}=-\frac{\Delta t}{2c}\alpha_{i}e_{i}\cdot g\beta \left(T-T_{0}\right), \end{array}$$

when *i* = 2 and *i* = 4, *α*_*i*_ = 1; otherwise, *α*_*i*_ = 0.

## 4. Results and Discussion

In this section, we will use the geometric model in Section 2 and the AH dynamics model in Section 3 to numerically simulate the AH flow of human eye with different morphological structures and physical properties and analyze the reasons of affecting the AH flow in the human eye.

### 4.1. AH Flow in the Normal Human Eye

In the normal human eye, the range of the rate of AH generation is 2.4 *μ*L/min to 3.0 *μ*L/min, [32] which is set to 2.5 *μ*L/min in this paper, the range of IOP is 15.5 ± 2.6 mmHg (1716 Pa~2407 Pa), [32] which is set to 1950 Pa in this paper. Other physical property parameters in the eye are set to a value within the normal range as shown in Table 1.

**Figure 5:**
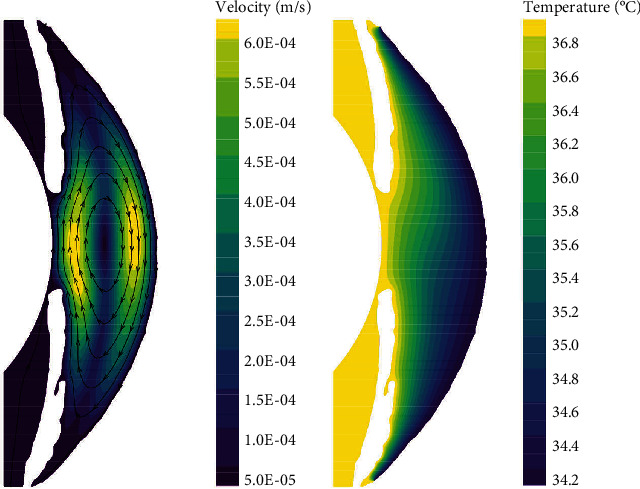
The flow of AH in the standing position of normal human eye. (a) Velocity distribution. (b) Temperature distribution.

In the normal human eye, the velocity distribution and temperature distribution of AH in the standing position are shown in Figure 5. From the velocity distribution diagram, it can be seen that the AH flows from the entrance of PC, then flows to the gap between the iris and the lens, and enters AC through the pupil. The AH in AC does natural convection under the temperature difference between the cornea and the other intraocular tissue, forming a clockwise vortex, and finally, the AH is discharged from TM. The maximum velocity of AH appears on the pupil axis, and it is about 6.47 × 10^−4^ m/s. It can be seen from the temperature distribution diagram that in the standing position, the temperature distribution decreases gradually from lens to cornea.

**Figure 6:**
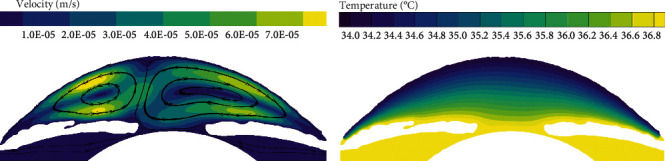
The flow of AH in the lying position of normal human eye. (a) Velocity distribution. (b) Temperature distribution.

The flow of AH in the lying position is shown in Figure 6, which is significantly different from the standing position. As can be seen from the velocity distribution diagram, the AH flows from PC forms two opposite vortices in AC. Meanwhile, the AH flows from the pupil to the cornea along the contact lines of the two vortices and then flows to the two AC angles, respectively. The maximum velocity is about 7.88 × 10^−4^ m/s. In the lying position, the temperature distribution of AH is basically symmetrical along the pupil axis.

### 4.2. The Influence of Morphological Structures

We selected human eye models with three different shapes of AC for simulation experiments. The experimental results are listed in Table 2.

From the velocity distribution diagram in Table 2, it can be found that in the human eyes with different AC shapes, the flow of AH in the lying position will form two opposite vortices in AC. In the theoretical model, the two vortices generated by AH flow are completely symmetrical with the same size and shape because the structure of AH is left-right symmetrical. However, in actual situations, the human eye has different structures and shapes, and the AC shape may not necessarily be a left-right symmetrical structure like the theoretical model. From the simulation results of the two AS-OCT images below, it can be seen that if the asymmetry of the AC is small, the flow of AH in AC will form two basically symmetrical vortices. If the asymmetry of AC is large, the flow of AH will form two different vortices in AC, and the maximum velocity of AH will appear in the smaller area of AC. This shows that the morphological structure of AC influences the flow of AH. If we use the theoretical model of AS to conduct numerical simulation, it may not be able to reflect the influence of AS shape on the flow of AH.

**Figure 7:**
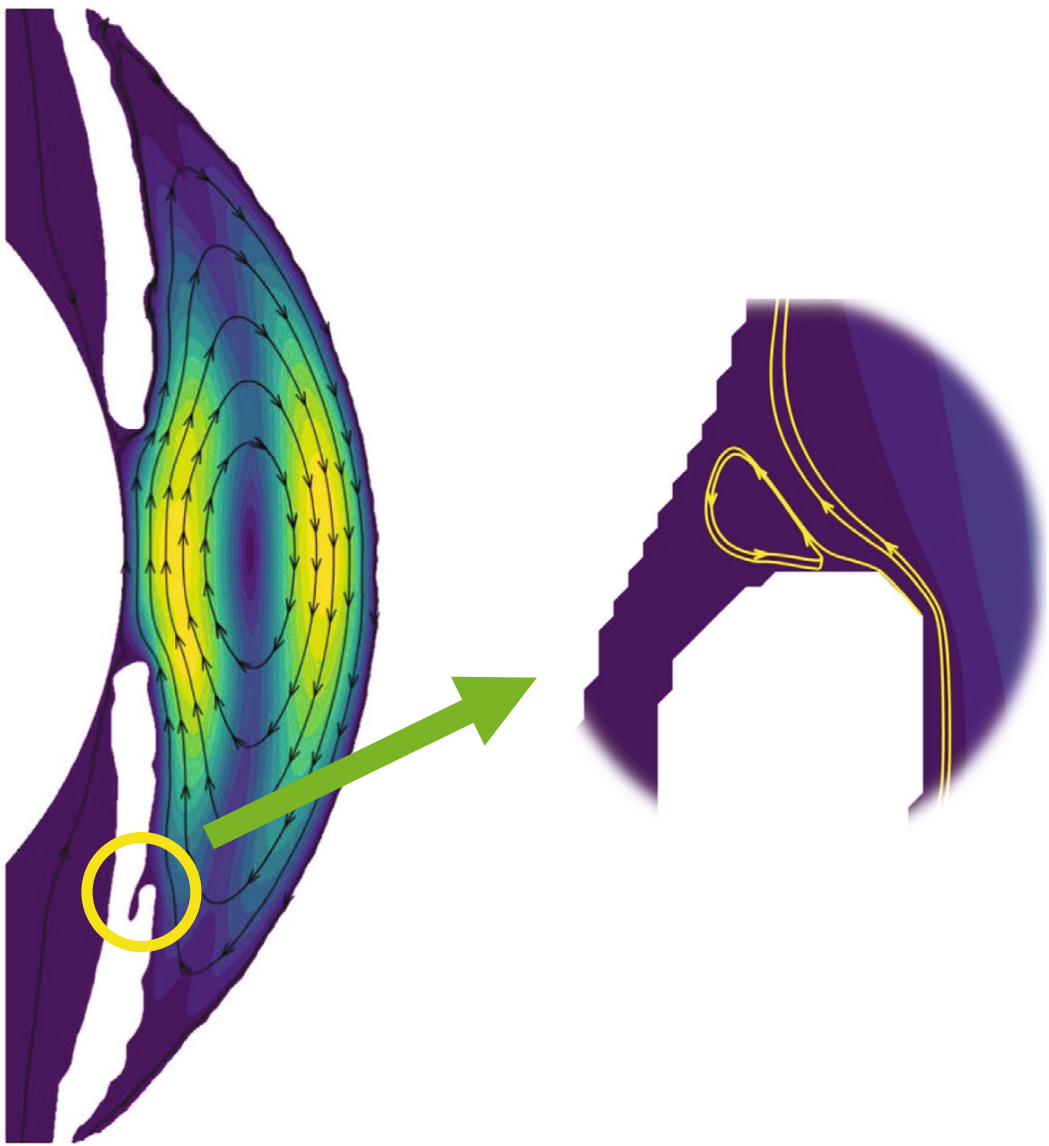
The vortices in the crypts of the iris.

At the same time, in the comparison between the theoretical AS geometric model and the geometric model based on the AS-OCT image, we found a unique phenomenon, and there are some small vortices in the iris crypts, as shown in Figure 7. In the theoretical AS geometric model, in order to facilitate the simulation, the iris surface is usually assumed to be a regular straight wall, which does not include information such as crypts; so, this phenomenon cannot be observed during the simulation. The geometric model based on the AS-OCT image contains these details. It is precise because of the existence of iris crypts that we can find the phenomenon. This finding also illustrates from the side that the geometric model based on the AS-OCT image can obtain more realistic and accurate results during simulation.

Previous studies have shown that when the cornea is deformed by the external forces, the structure of AC will change, which will affect the flow of AH in AC. One of the more common situations is that long-term wearing of an orthokeratology lens in the treatment of myopia causes the shape of the cornea to change, and the central part of the cornea becomes flat. In order to simulate this phenomenon, we assumed that in the normal eye, the cornea is deformed under the influence of external force, and a flat indentation with a distance of *d* is formed in the center. We simulated the AH flow within the indentation distance of 0.3 mm -0.5 mm, and the experimental results were listed in Table 3.

From the results in Table 3, we can see that with the increase of the cornea indentation distance, the flow of AH does not change significantly, but the maximum velocity of AH is gradually decreasing, which is more consistent with the conclusions of the literature. In the change from 0.3 mm to 0.5 mm, the maximum velocity of AH is reduced by 17%. Compared with the maximum velocity in the normal human eye, it drops more. The reason for this phenomenon may be that the inlet velocity of the aqueous humor remains unchanged; that is, the flow of aqueous humor per unit time remains unchanged, but the volume of the anterior chamber decreases with the increase of the corneal deformation distance. Like the flow in the pipe, the flow rate per unit time remains unchanged, and the smaller the volume, the greater the flow velocity. This may have a certain influence on the normal discharge of AH from TM. Subsequent studies should also consider the influence of other deformed states of the cornea on the flow of AH.

There are also some researches to study the influence of pupil size on the AH flow by setting different sizes of pupil. In this paper, numerical simulation experiments were carried out on human eyes with pupil sizes of 2.76 mm, 3.69 mm, and 4.91 mm, respectively. The results show that the velocity distribution and temperature distribution of AH do not change significantly, there is no obvious linear relationship between the pupil size and the velocity of AH, and the size of pupil has little effect on the flow of AH.

### 4.3. The Influence of Physical Properties

**Figure 8:**
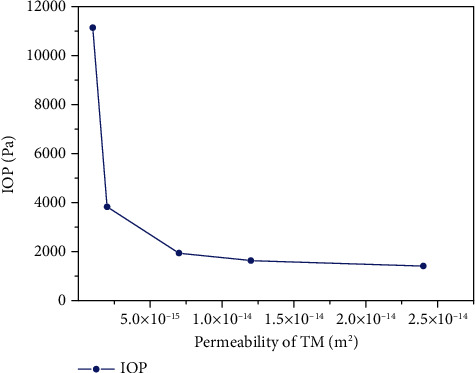
IOP changes with the permeability of TM.

In angle closure glaucoma, the iris bombe blocks the normal outflow of AH, leading to the increase of IOP, which belongs to the influence of morphological changes on the flow of AH. In open angle glaucoma, the AC angle is always open, and the outflow of AH is blocked by the TM, which is due to the lesions of the physical characteristics of the TM. Based on the numerical simulation of the normal human eye, we change the permeability of the TM and keep other parameters unchanged to simulate the flow of AH. When the permeability of the TM changes, the IOP will also change, and the flow of AH will reach the new balance. The result is shown in Figure 8.

As can be seen from Figure 8, the IOP decreases with the increase of TM permeability, but the degree of IOP decreases is not linear. When the permeability of TM is lower than 5.0 × 10^−15^ m^2^, the IOP decreases very quickly with the increase in permeability. When the permeability of TM is higher than this value, as the permeability increases, the IOP decreases gently with the increase of permeability. This shows that IOP can be effectively reduced by drugs that increase TM permeability.

**Figure 9:**
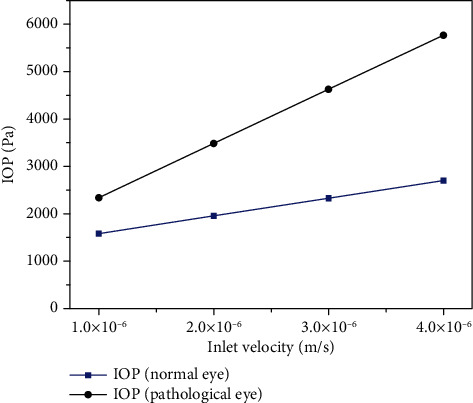
IOP changes with the secretion rate of AH.

Many studies have proved that IOP can be effectively controlled by controlling the secretion rate of AH. In this paper, we set up two groups of human eye models, normal eyes (TM permeability *K* = 7.0 × 10^−15^ m^2^) and pathological eyes ( *K* = 2.3 × 10^−15^m^2^), and observed the changes of IOP in different eyes by changing the secretion rate of AH in two models. The simulation results are shown in Figure 9. Under normal circumstances, the secretion rate of AH is 2.5 *μ*L/min, and after conversion, the inlet velocity is 2.0 × 10^−6^ m/s.

As can be seen from Figure 9, with the increase of AH secretion rate, the IOP in both groups increased, but the degree of IOP increases in pathological eyes is significantly higher than that in normal eyes. Therefore, under the pathological conditions, high IOP can be effectively controlled by reducing the secretion rate of AH with drugs.

**Figure 10:**
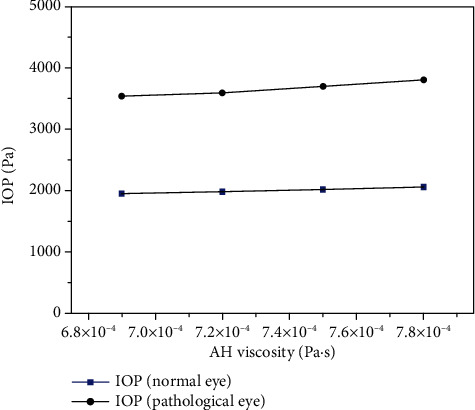
IOP changes with the viscosity of AH.

The viscosity coefficient of fluid is also a physical characteristic parameter closely related to fluid flow, but there are few studies on the relationship between the AH viscosity and the AH flow. In order to explore the relationship between the two, we studied the changes of IOP under different viscosity coefficients by changing the viscosity of AH. The result is shown in Figure 10.

From the result, we can see that compared with the permeability of TM and the secretion rate of AH, IOP is not sensitive to the changes in AH viscosity, which may be one of the reasons why it is not considered as a factor to control IOP.

## 5. Conclusions

This paper denoised and segmented the AS-OCT images and combined the structure data of the human eye to establish the geometric model of AS which contains individual characteristics. At the same time, using the advantages of the LB method in the study of complex fluid motion problems, the LB model of AH dynamics including the processes of AH flow, heat transfer, and outflow of TM was established. On this basis, we simulated and analyzed the AH flow of human eyes with different morphological structures and physical properties and obtained the following results:

1. The velocity distribution and temperature distribution of AH flow in the human eye were plotted. The velocity distribution and temperature distribution of AH in standing position and lying position are significantly different
2. The morphological structures of the intraocular tissues will influence the flow of AH. The shape of AC will significantly change the flow pattern of AH in AC. In the asymmetrical AC, the AH will form two different vortices. The crypts of iris will influence the AH flow on the iris surface. The indentation of cornea will also influence the AH flow. In general, the greater the indentation distance of cornea, the smaller the maximum velocity of AH. The size of pupil has no obvious influence on the AH flow
3. The physical properties of the intraocular tissues will influence the flow of AH. The IOP will decrease with the increase of the TM permeability and will increase with the increase of the AH secretion rate; so, we can control the IOP by changing the permeability of TM or the secretion rate of AH with medicines. In addition, The IOP is not sensitive to changes in the viscosity of AH

Compared with experiments, the method in this paper can obtain some physiological and pathological parameters that are difficult to directly measure, and compared with the theoretical models, the method has the better authenticity and accuracy in the results of numerical simulation. Our next step is to continuously improve the model and method in order to obtain more valuable results and make these conclusions more rigorous and practical.

## Figures and Tables

**Table 1:** Physical property parameters in normal human eyes.

Parameter	Value
AH density, *ρ*	9.93 × 10^2^kg/m^3^ [9, 33]
AH dynamic viscosity coefficient, *μ*	6.97 × 10^−4^ Pa∙s [33]
AH thermal conductivity, *D*	0.58W/m∙°C [9]
TM permeability, *K*	7.0 × 10^−15^ m^2^ [34]
Prandtl number, Pr	5.03
Relaxation time, *τ*	1.0

**Table 2:** The influence of AC shape on the flow of AH.

AS-OCT image	AC shape	Velocity distribution
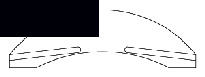	Completely symmetrical	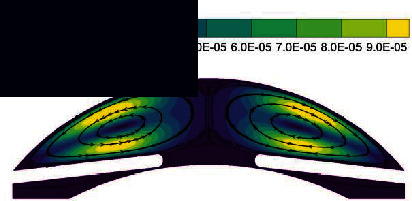
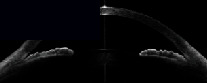	Basically symmetrical	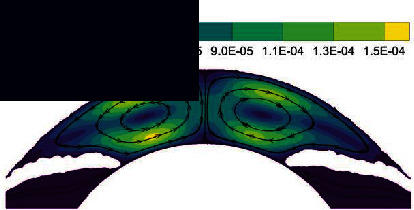
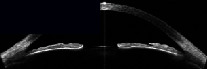	Asymmetrical	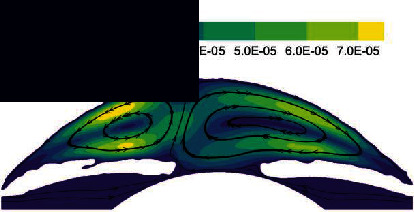

**Table 3:** The influence of cornea indentation on the flow of AH.

The distance of cornea indentation	0.3 mm	0.4 mm	0.5 mm
Velocity distribution	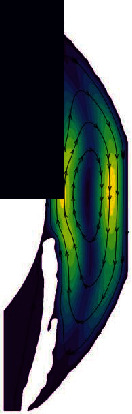	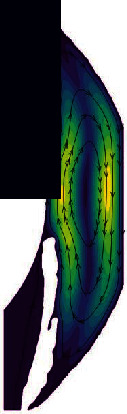	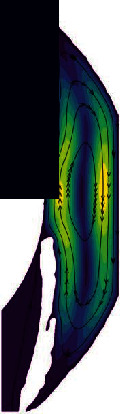
The maximum velocity of AH	5.8 × 10^−4^ m/s	5.2 × 10^−4^ m/s	4.8 × 10^−4^ m/s

## Data Availability

Data sharing does not apply to this article as no datasets were generated or analyzed during the current study. All data generated or analyzed during this study are included in this published article.
